# Comparative analysis of the vaginal microbiome in health and disease

**DOI:** 10.1186/gb-2011-12-s1-i17

**Published:** 2011-09-19

**Authors:** Bryan A White, Andres M Gomez, Mengfei Ho, Margret Berg Miller, Susan M Thomas, Carl J Yeoman, Suleyman Yildirim, Douglas J Creedon, Tony L Goldberg, Steven R Leigh, Karen E Nelson, Rebecca M Stumpf, Brenda A Wilson

**Affiliations:** 1 The Institute for Genomic Biology, University of Illinois, Urbana, IL 61801, USA; 2 Department of Animal Sciences, University of Illinois, Urbana, IL 61801, USA; 3 Division of Biomedical Sciences, University of Illinois, Urbana, IL 61801, USA; 4 Department of Microbiology, University of Illinois, Urbana, IL 61801, USA; 5 Department of Obstetrics and Gynecology, Mayo Clinic, Rochester, MN 55905, USA; 6 Department of Pathobiological Sciences, University of Wisconsin, Madison, WI 53706, USA; 7 Department of Anthropology, University of Illinois, Urbana, IL 61801, USA; 8 J. Craig Venter Institute, Rockville, MD 20850, USA

## 

Diseases of the vaginal tract result from perturbations of the complex interactions among microbes of the host vaginal ecosystem. Recent advances in our understanding of these complex interactions have been enabled by next-generation-sequencing-based approaches, which make it possible to study the vaginal microbiome. In harnessing these approaches, we are beginning to define what constitutes an imbalance of the vaginal microbiome and how such imbalances, along with associated host factors, lead to infection and disease states such as bacterial vaginosis (BV), preterm births, and susceptibility to HIV and other sexually acquired infections. We have exploited various approaches to this end: comparative analysis of reference microbial genomes of vaginal isolates; comparative microbiome, metabolome and metagenome analysis of vaginal communities from subjects deemed to be healthy and individuals with BV; and comparative microbiome analysis of vaginal communities from humans and non-human primate species. The results from comparative genome sequencing have led us to suggest that different strains of the proposed pathogen *Gardnerella vaginalis* have different virulence potentials and that the detection of *G. vaginalis* in the vaginal tract is not indicative of a disease state [1]. Comparative microbiome, metabolome and metagenome analysis of vaginal communities from humans has demonstrated that the microbial communities from subjects with BV have a defined bacterial composition and metabolic profile that is distinct from subjects who do not have BV [2 and unpublished observations]. Our studies of microbial communities from non-human primate species and humans provide a unique comparative context. From an evolutionary perspective, humans and non-human primates differ considerably in mating habits, estrus cycles and gestation period. Moreover, birth is difficult in humans relative to other primates, increasing the risks of maternal injury and infection. In light of these numerous differences between humans and non-human primates, we hypothesize that humans have microbial populations that are distinct from those of non-human primates. Preliminary results show that the vaginal microbiomes of non-human primates are more diverse and are compositionally distinct from human vaginal microbiomes [3,4]. The composition of bacterial genera found in non-human primates is dissimilar to that seen in humans, most notably with lactobacilli being much less abundant in non-human primates. Our observations point to vaginal microbial communities being an important component of an evolutionary set of adaptations that separates humans from other primates and is of fundamental importance to health and reproductive function.
